# Brain Activation by Peptide Pro-Leu-Gly-NH_2_ (MIF-1)

**DOI:** 10.1155/2010/537639

**Published:** 2010-03-28

**Authors:** Reas S. Khan, Chuanhui Yu, Abba J. Kastin, Yi He, Rudolph H. Ehrensing, Hung Hsuchou, Kirsten Prufer Stone, Weihong Pan

**Affiliations:** ^1^Blood-Brain Barrier Group, Pennington Biomedical Research Center, Baton Rouge, LA 70808, USA; ^2^The Ochsner Clinic Foundation, New Orleans, LA 70121, USA

## Abstract

MIF-1 (Pro-Leu-Gly-NH_2_) is a tripeptide for which the therapeutic potential in Parkinson's disease and depression has been indicated by many studies. However, the cellular mechanisms of action of MIF-1 are not yet clear. Here, we show the specific brain regions responsive to MIF-1 treatment by c-Fos mapping, and determine the kinetics of cellular signaling by western blotting of pERK, pSTAT3, and c-Fos in cultured neurons. The immunoreactivity of c-Fos was increased 4 hours after MIF-1 treatment in brain regions critically involved in the regulation of mood, anxiety, depression, and memory. The number of cells activated was greater after peripheral treatment (intravenous delivery) than after intracerebroventricular injection. In cultured SH-SY5Y neuronal cells, c-Fos was induced time- and dose-dependently. The activation of cellular c-Fos was preceded by a transient increase of mitogen-activated protein kinase pERK but a reduction of phosphorylated Signal Transducer and Activator of Transcription (pSTAT3) initially. We conclude that MIF-1 can modulate multiple cellular signals including pERK, and pSTAT3 to activate c-Fos. The cellular activation in specific brain regions illustrates the biochemical and neuroanatomical basis underlying the therapeutic effect of MIF-1 in Parkinson's disease and depression.

## 1. Introduction

Melanocyte-stimulating hormone release inhibiting factor-1 (MIF-1), also known as PLG based on its amino acid structure (Pro-Leu-Gly-NH_2_), is an endogenous brain peptide that exerts a variety of pharmacological effects on the central nervous system [1]. Clinical studies have shown that MIF-1 can alleviate symptoms in Parkinson's disease (PD) and mental depression. These have been summarized elsewhere, although the review overlooked a report in the Chinese literature of favorable results of MIF-1 in PD [2]. MIF-1 was the first hypothalamic peptide shown to act ‘‘up” on the brain, not just ‘‘down” on the pituitary [3]. In human plasma, a duration of 5 days is required for 50% degradation of MIF-1 at 37°C [4]. This remarkable stability of MIF-1 in human blood, coupled with its persisting biological activity, makes MIF-1 a worthwhile candidate as a human therapeutic agent. 

The therapeutic potential of MIF-1 indicates the importance of further determining how it acts on the brain. There already is evidence that MIF-1 activates CNS pathways related to opiate and dopaminergic systems based on the following evidence: (1) MIF-1 antagonizes opiate actions, and the first report of such activity correctly predicted the discovery of other endogenous antiopiate peptides blocking the analgesic effect of morphine and enkephalin in a radiant heat tail-flick assay [5]. MIF-1 also significantly antagonized the effects of morphine in a double-blind study in humans [6]. (2) MIF-1 is effective in the DOPA-potentiation, oxotremorine antagonism, and deserpidine antagonism tests [7]. In a small number of patients with PD, Barbeau [8] found its potentiation of the effects of levodopa to be remarkable; (3) MIF-1 can modulate dopaminergic transmission in-vitro as well as in-vivo, increasing the binding affinity of agonists to the high-affinity state of the dopamine receptor and shifting the ratio of high- and low-affinity states of the dopamine receptor in favor of the G-protein-coupled high-affinity state [9]. (4) The actions of MIF-1 appear rather selective toward dopamine receptors since it does not interact with other aminergic receptors such as adrenergic [9], GABAergic [10], or serotonergic receptors [11]. (5) MIF-1 also facilitates passive acquisition of brightness discrimination, passive avoidance retention, appetitive maze performance, and inhibits shock-suppressed water intake, findings interpreted as contributing to the processes of memory consolidation [1, 12]. However, MIF-1 does not change cAMP in the brain in contrast to the increase induced by *α*-MSH, and its induction of cGMP is not robust in rat brain [13]. This led us to study alternative cellular signaling pathways.

A number of exogenous stimuli, including an increase in neuronal activity, trigger the transcription of c-Fos, which is an immediate early gene that in combination with the specific Jun proteins forms the heterodimeric transcription regulator, AP-1 [14]. The extracellular signal-regulated kinase (ERK)-1/2 pathways are involved in the induction and regulation of c-Fos [15]. In this study, we performed a brain mapping of the neuronal expression of c-Fos after intravenous (iv) or intracerebroventricular (icv) injection of MIF-1. We also determined the upstream signaling pathways leading to the activation.

## 2. Materials and Methods

### 2.1. Animals and Reagents

All studies were conducted following a protocol approved by the Institutional Animal Care and Use Committee. Adult male C57BL/6J mice were purchased from Jackson Laboratories (Bar Harbor, ME) and housed in the animal care facility for at least 2 weeks before study. The mice were housed 4/cage and provided with regular rodent chow and water *ad libitum*. The ambient room temperature was 23°C. The light span was 07:00–19:00 hours in a light-dark (LD) 12:12 lighting regimen. 

 MIF-1 was purchased from Phoenix pharmaceuticals (Burlingame, CA) and reconstituted in phosphate-buffered saline (PBS) before use. Rabbit polyclonal anti-c-Fos antibody, goat anti-pSTAT3-Tyr^705^ antibody, and mouse anti-pERK E-4 monoclonal antibody were purchased from Santa Cruz Biotechnology (Santa Cruz, CA). Anti-rat NeuN antibody was purchased from Chemicon (Billerica, MA). Anti-*β*-actin antibody and other chemicals were obtained from Sigma (St. Louis, MO).

### 2.2. Immunofluorescent Staining

In accordance with a protocol approved by the Institutional Animal Care and Use Committee, C57BL/6J male mice (5–7 weeks old) were anesthetized by intramuscular injection of ketamine and xylazine. MIF-1 was delivered either into the isolated left jugular vein (iv) at 10 *μ*g/mouse in a volume of 50 *μ*L (*n* = 3), or into the right lateral cerebral ventricle (icv) at 1 *μ*g/mouse in a volume of 1 *μ*L (*n* = 3). The control groups received the same volume of PBS (*n* = 3). The icv coordinates were 2 mm lateral and 0.2 mm posterior to the bregma, and 2.5 mm below the skull. The mice were perfused intracardially with 30 mL of normal saline (NS) followed by 60 mL of 4% paraformaldehyde. The brain was postfixed overnight in 4% paraformaldehyde and cryoprotected in 15% and then 30% sucrose. Coronal sections of 20 *μ*m thickness were obtained by the use of a cryostat. The tissue sections were permeabilized with 0.3% Triton X-100 and blocked with 10% normal donkey serum, and incubated with a primary antibody overnight at 4°C. The antibodies include rabbit polyclonal anti-c-Fos antibody (1  :  200, Santa Cruz Biotechnology sc-52) and anti-rat NeuN antibody (1  :  100, Chemicon). After thorough washing, they were incubated with respective Alexa488-conjugated secondary antibodies at room temperature for 1 hour, washed, and mounted. Negative controls were incubated with a secondary antibody only.

### 2.3. Cell Culture and Western Blotting

SH-SY5Y human neuroblastoma cells (American Type Culture Collection, ATCC, Manassas, VA) were grown in Dulbecco's modified Eagle's medium (DMEM) with 10% fetal bovine serum (FBS). The cells were differentiated by treatment with 10 *μ*M of all-trans retinoic acid (Sigma, St. Louis, MO, USA) between 1 and 6 days after plating. Sixteen hours after serum starvation, the cells were treated with MIF-1 (1–10 ng/mL for different time intervals (0–3 hours). All cells were plated at the same time and treated according to the time intervals designed for individual experiments. The cells were lysed in ice-cold RIPA buffer (100 mM NaCl, 10  mM Tris, pH 7.2, 0.1% SDS, 1% Triton X-100, 1% deoxycholate, 5 mM EDTA) in the presence of protease inhibitor cocktail (Pierce, Rockford, IL). The lysates were sonicated and cleared by ultracentrifugation. The protein content was measured by bicinchoninic acid assay (Pierce). Thirty to 50 *μ*g of protein was electrophoresed on 12% SDS-polyacrylamide gel and transferred to a nitrocellulose membrane (Bio-Rad, Hercules, CA). The membrane was blocked with 5% non-fat dry milk in Tris-buffered saline (pH 7.6) containing 0.1% Tween-20, and probed with rabbit anti-c-Fos (polyclonal, 1  :  200, Santa Cruz Biotechnology, sc-52), goat anti-pSTAT3 Tyr 705 (polyclonal, 1  :  1,000 sc-7993), mouse anti-pERK E-4 (monoclonal, 1  :  500, sc-7383 and mouse anti-*β*-actin (monoclonal, 1  :  10,000, Sigma, A2228) overnight at 4°C. After thorough washing, the membranes were incubated with horseradish peroxidase-conjugated secondary antibody for 1 hour at room temperature. The signals were developed with enhanced chemiluminescence-plus western blotting detection reagents (Amersham Biosciences, Piscataway, NJ).

## 3. Results

### 3.1. MIF-1 Increases c-Fos Expression in Specific Different Brain Regions

**Figure 1:**
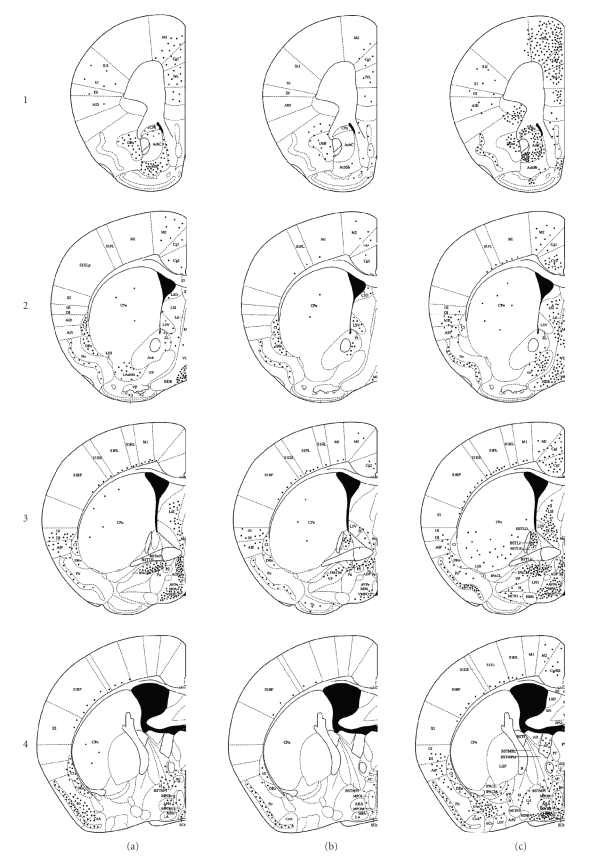

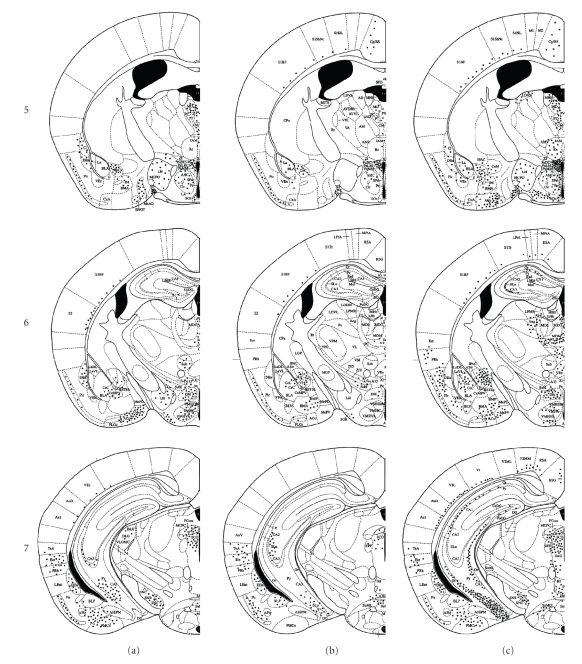
Mapping of MIF-1-induced brain activation of c-Fos immunoreactivity and icv and iv administration. Vertical panel (a) represents the control brain, panel (b) represents c-Fos immunoreactivity 4 hours after icv injection of MIF-1 (1 *μ*g/mouse), and panel (c) represents c-Fos immunoreactivity 4 hours after iv injection of MIF-1 (10 *μ*g/mouse). Horizontal rows 1–7 represent various sections of the brain from the anterior to the posterior part of the brain. The distances from interaural and bregma in mm are 1: 5.58 and 1.78, 2: 4.54 and 0.74, 3: 4.06 and 0.26, 4: 3.46 and −0.34, 5: 5.58 and 1.78, 6: 2.34 and −1.46, 7: 1.10 and −2.70.

**Figure 2:**
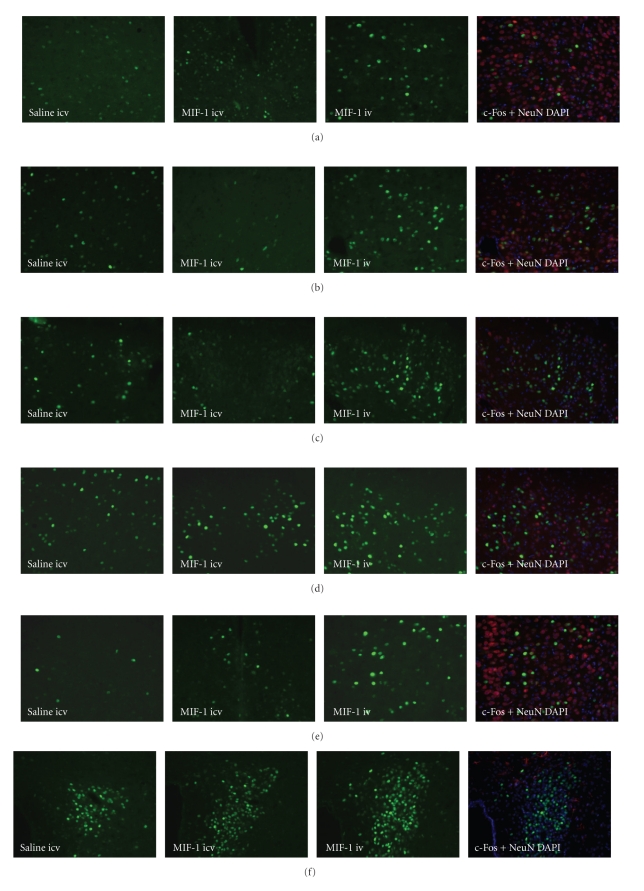

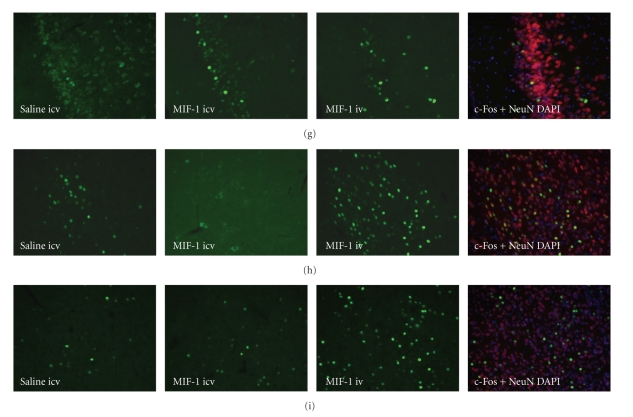
Detailed immunohistochemical representation of MIF-1 induced brain activation by c-Fos immunoreactivity. The panels on the left show basal c-Fos activity in different regions. The second panels show c-Fos immunoreactivity 4 hours after iv injection of MIF-1 (10 *μ*g/mouse). The third panels show c-Fos immunoreactivity 4 hours after icv injection of MIF-1 (1 *μ*g/mouse). The last panels show NeuN (+) neurons (red), DAPI-labeled nucleus (blue), as well as c-Fos (green) in sections after iv injection. (a) Cingulate cortex, (b) Thalamic nucleus, (c) Paraventricular thalamic nucleus, anterior part, (d) Paraventricular thalamic nucleus, posterior part, (e) Infralimbic cortex, (f) Paraventricular nucleus of the hypothalamus, (g) medial amygdaloid nucleus, (h) Fiber tract in piriform cortex, and (i) Nucleus accumbens.

We previously observed that MIF-1 is saturably transported from blood to brain [16]. In this study, we demonstrate that iv injection of MIF-1 increased c-Fos immunoreactivity in different brain regions. The regions that showed the highest c-Fos activation include cingulate cortex (Figures 1(c3) and 2(a)), infralimbic cortex (Figures 1(c1) and 2(e)), nucleus accumbens (Figures 1(c1) and 2(i)), paraventricular nucleus (PVN) in the hypothalamus (Figures 1(c5) and 2(f)), medial basal amygdaloid nucleus (Figures 1(c6) and 2(h)), fiber tract in piriform cortex (Figures 1(c6) and 2(g)), paraventricular thalamic nucleus (Figures 1(c6), 2(c), and 2(d)), and other thalamic nuclei (Figures 1(c7) and 2(b)). Double labeling with NeuN showed that many of the cells expressing c-Fos were neurons. The increase in c-Fos expression was greater after iv injection than after icv injection in many brain regions. This is consistent with the ability of stable substances in the cerebral circulation to reach all parts of the brain rapidly. After icv, the increase of c-Fos was mainly seen in the paraventricular nucleus of the hypothalamus (Figures 1(b5) and 2(f)). The basal level of activation of PBS icv and iv was similar. Thus, only the icv-injected controls are shown in Figure 2. Table 1 summarizes the number of c-Fos immunopositive cells in different brain regions.

### 3.2. MIF-1 Increases c-Fos Expression in Cultured Neurons

**Figure 3:**
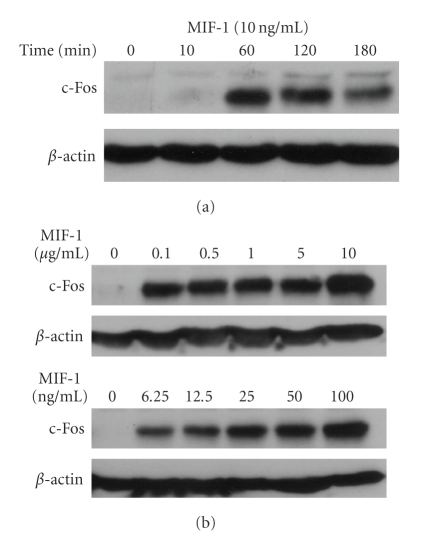
MIF-1 activates cultured SH-SY5Y neurons, shown by western blotting of c-Fos. (a) MIF-1 increased neuronal c-Fos at 1, 2, and 3 hours at a concentration of 10 ng/mL, showing its time-dependent pattern. (b) MIF-1 increased c-Fos at all concentrations tested at 1 hour, showing its dose-response.

Western blotting showed that the c-Fos signal was increased 60 minutes after MIF-1 treatment (10 ng/mL). This persisted at 2 and 3 hours (Figure 3(a)). There was also a dose-dependent increase, highest at the maximal dose of 10 *μ*g/mL, but even the lowest concentration of MIF-1 tested (6.25 ng/mL) showed a robust induction of c-Fos 1 hour later (Figure 3(b)). 

### 3.3. MIF-1 Induces a Transient Increase of pERK but Causes a Biphasic Change of pSTAT3

**Figure 4:**
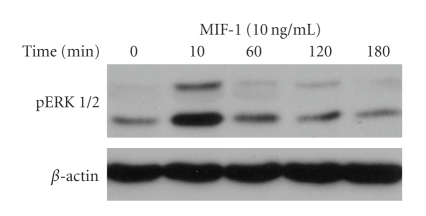
MIF-1 (10 ng/mL) induced a time-dependent increase of pERK1 (44 kD) and pERK2 (42 kD), most apparent at 10 minutes.

**Figure 5:**
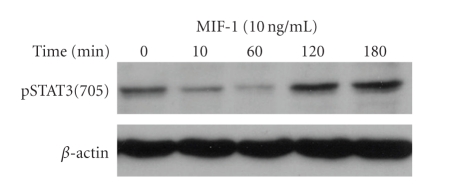
MIF-1 (10 ng/mL) induced a time-dependent change of pSTAT3-Y^705^, shown by an initial decrease at 10 and 60 minutes, and a subsequent increase at 2 and 3 hours.

Western blotting analysis showed that pERK expression was increased in SH-SY5Y cells 10 minutes after MIF-1 treatment (10 ng/mL) (Figure 4). By contrast, the cells responded to MIF-1 with an initial reduction of pSTAT3 at 10 and 60 minutes, followed by increased pSTAT3 expression at 2 and 3 hours (Figure 5).

## 4. Discussion

In this study, we showed that MIF-1 induces c-Fos activation in different regions of the brain and in cultured neurons. MIF-1 also induced c-Fos in non-NeuN (+) cells. This is the first report stating that MIF-1 increases the phosphorylation of ERK, indicative of activation of a mitogen-activated protein kinase (MAPK) pathway. Our results show that MIF-1 decreased pSTAT3 initially (10 and 60 minutes), but then increased pSTAT3 at later times (2 and 3 hours) in neuronal culture, probably reflecting a secondary mechanism. This also is the first report showing that MIF-1 modulates pSTAT3 activation, although the functional consequences are not clear. These are not classical elements of the GPCR signaling pathway, and suggest a broader action of this orphan ligand. 

While a peptide like MIF-1 may either show direct CNS effects or activate secondary mediators, c-Fos immunoreactivity reflects a final common pathway of brain activation as a result of MIF-1 application. The immediate early gene product c-Fos is an easily identifiable and rather sensitive marker for CNS activation. The increase of c-Fos has been demonstrated after various stimuli, including growth factors, ion channel activation, neurotransmitter release, and behavioral modifications [17]. The increase of its expression can be mediated by many intracellular signaling pathways, such as increases in cAMP, calcium influx, and activation of MAPK [18]. Our results show that the increase in c-Fos expression is greater after iv injection than after icv injection in many brain regions. After icv administration, the increase of c-Fos immunoreactivity was mainly seen in the PVN of the hypothalamus. Considering that less than 1% of MIF-1 from blood permeates the blood-brain barrier to reach the brain, like most centrally active peptides and even L-DOPA and morphine, this indicates that blood-borne MIF-1 is more effective than when administered into the slower moving ventricular system of the brain. This is not surprising considering that no neuron is more than about 8 *μ*m from a capillary [19]. It is also possible that peripherally injected MIF-1 activates additional mediators, thus triggering a cascade of signalling events. The implications of MIF-1-induced c-Fos activation in different brain regions are discussed below.

(1) Cingulate cortex: the cingulate cortex is an anatomically and functionally heterogeneous region and it modulates emotion and mood. Attention deficit in PD has been explained by dopamine depletion of the cingulate cortex [20]. Fornix-induced lesions can reduce c-Fos immunoreactivity in the cingulate cortex of rats [21]. Earlier studies showed that lesions of the cingulate cortex affect behavior in the open-field and interspecies aggression behavior in rats [22]. Melatonin (5-methoxy-N-acetyltryptamine) was reported to be critically involved in the regulation of both mood and pain [23]. Melatonin receptor type 1 (MT1)-knockout mice display depression-like behavior with altered sensory responses and attention deficits [24]. These previous studies show that the central melatoninergic system might play an important role in the mechanism of interactions between pain and depression, and the anterior cingulate cortex could be a forebrain region of interest in this process. MIF-1 can potentiate the melanocyte-lightening effect of melatonin in rats [25], and its effects in patients with PD are associated with marked mood elevation. Therefore, the MIF-1-induced c-Fos activation in the cingulate cortex supports the speculation that the effectiveness of MIF-1 in treating movement disorders may be associated with increased melatonin secretion. 

(2) Thalamus: the lateral geniculate nucleus of the thalamus is involved in fluctuations in both visual attention and visual awareness [26]. MIF-1 can facilitate acquisition of brightness discrimination, probably by affecting processes of attention when rats are tested with a spatial extradimensional shift problem after acquisition of a visual task [27]. c-Fos expression has been found in the medial geniculate body (MGB) of the thalamus after mice acquired a visually cued conditioned fear. The activation of c-Fos by MIF-1 in the thalamus supports the direct effects of MIF-1 there. 

(3) Infralimbic cortex: the infralimbic cortex (IL) is a cortical region in the medial prefrontal cortex that is important in tonic inhibition of subcortical structures and emotional responses such as fear [28]. Electrical stimulation of the IL reduces conditioned fear and strengthens extinction memory. This illustrates cortical control over extinction processes, which is one of the simplest forms of emotional regulation [29]. Less c-Fos immunoreactivity is present in the IL of the extinction-resistant mouse compared with the control [30]. In a 12-choice Warden maze for a palatable food reward, rats receiving MIF-1 have shorter latencies and make fewer errors than controls during learning, but not extinction, of the task [31]. Our results provide the first direct evidence that MIF-1 can activate IL neurons.

(4) Nucleus accumbens: both typical and atypical antipsychotic drugs increase c-Fos protein expression in the nucleus accumbens shell [32]. The ability of MIF-1 to increase c-Fos in the forebrain may have considerable predictive validity for antipsychotic drug actions. Administration of MIF-1 to patients with depression showed substantial improvement within a few days after initiation of treatment [33–35]. Previous studies showed that antipsychotic drugs like clozapine and amperozide preferentially increase dopamine release in the rat nucleus accumbens and prefrontal cortex [36]. Hippocampal dopamine receptors modulate c-Fos expression in the rat nucleus accumbens that is evoked by chemical stimulation of the ventral hippocampus [37]. Thus, MIF-1 may activate cells in the nucleus accumbens and modulate dopaminergic activity there to alleviate mood and schizoaffective disorder.

(5) PVN: the PVN plays important roles in neuroendocrine and autonomic nervous system controls. For example, many experimental challenges (such as restraint stress, dehydration, and immune challenge) induce c-Fos expression in the PVN [38, 39]. Although MIF-1 has no effects on a naloxone-sensitive peptide YY (PYY) model of hyperphagia after PYY injection into the PVN [40], the induction of c-Fos by MIF-1 through both iv and icv injection indicates that PVN is a major site of action for MIF-1. 

(6) Medial amygdaloid nucleus: an avoidance task in the elevated T maze can increase Fos protein expression in the medial amygdaloid nucleus. The amygdala is a point of convergence for conditioned and unconditioned stimuli and seems to impart emotional value to sensory stimulation [41]. Increased expression of immediate early genes in the amygdala has been reported in several paradigms of aversive conditioning [42]. These findings may provide a physiological basis for MIF-1 induced c-Fos activation in the amygdala.

(7) Piriform cortex: the presence of the c-Fos protein has been shown in the piriform cortex at different stages during the acquisition of trace conditioning in rabbits. MIF-1 also facilitates passive avoidance retention and inhibits shock-suppressed water intake, findings interpreted as contributing to the processes of memory consolidation [12]. In elevated-plus-maze tests, mice treated with Tyr-MIF-1 tend to spend more time in the open arms compared with the control group, suggesting the anxiolytic properties of this peptide that shows structural homology with MIF-1 [43]. Training induces c-Fos mRNA expression in the piriform cortex and the neocortex of n-3-fatty acid depleted rats, who showed learning improvement in an olfactory discrimination task [44]. MIF-1 also improves the capacity of rats to store information received through olfactory cues in social investigatory behavior [45].

In summary, the results show that MIF-1 increased c-Fos expression in brain regions involved in the regulation of mood, anxiety, depression, and memory. Blood-borne MIF-1 induced a greater extent of activation than that after icv, suggesting a prominent role of blood-brain barrier permeation. The activation status of pERK and pSTAT3 may contribute to the overall expression level of c-Fos. Thus, the results indicate the cellular mechanisms of actions of MIF-1 that may underlie the therapeutic effects of MIF-1 in the treatment of Parkinson's disease and depression.

## Figures and Tables

**Table 1:** Relative numbers of c-Fos (+) cells after MIF-1 treatment.

Structure abbreviation	Figure 1 diagram	Structure	Saline	icv	iv
cg1	(1)(a)–(c)	Cingulate cortex, area1	+^a^	+^a^	+++++^c^
cg2	(2)(a)–(c)	Cingulate cortex, area2	+^a^	+^a^	++++^d^
IL	(1)(a)–(c)	Infralimbic cortex	+	o	++^b^
DEn	(1)(a)–(c)	Dorsal endopiriform cortex	+++^b^	++	+++^d^
CPu	(1)(a)–(c)	Caudate, putamen	+	o	++^d^
AcbC	(1)(a)–(c)	Accumbens nucleus, core	+	+	+++++^d^
Pir	(3)(a)–(c)	Piriform cortex	++^b^	++^b^	+++^b^
PaDC	(5)(a)–(c)	Paraventricular hypothalamic nucleus, dorsal cap	+++	+++	+++++^d^
PaLM	(5)(a)–(c)	Paraventricular hypothalamic nucleus, lateral magnocellular	+++	+++	+++++^d^
PV	(6)(a)–(c)	Paraventricular thalamic nu	+++	+	+++++^d^
PCom	(7)(a)–(c)	Nucleus of posterior commissure	++^b^	+	++^b^
MCPC	(7)(a)–(c)	Magnocellular nucleus of posterior commissure	+	o	++^b^
RPF	(7)(a)–(c)	Retroparafascicular nucleus	o	+	++^b^
PAG	(7)(a)–(c)	Periaqueductal gray	+++^d^	++^b^	+++++^d^
IMD	(6)(a)–(c)	Intermed-dorsal thalamic nucleus	+++^d^	++^b^	+++++^d^
BMA	(6)(a)–(c)	Basomedial amygdaloid nucleus, anterior	+	++^b^	+++^c^

o: absent immunoreactivity; +: low immunoreactivity; ++: modest immunoreactivity; +++: moderate immunoreactivity; ++++: dense immunoreactivity; +++++: very dense immunoreactivity. a: punctate immunoreactivity; b: sparse immunoreactivity; c: variable density; d: cluster of dense immunoreactivity.
